# Liposomal Bupivacaine via an Adductor Canal Block Compared to a Peripheral Nerve Catheter and No Block After Total Knee Arthroplasty: A Retrospective Analysis

**DOI:** 10.7759/cureus.66891

**Published:** 2024-08-14

**Authors:** Dakota Harvey, Andrew Chafin, Michael Kazior, Amol M Karmarkar, Charmi Kanani, Brooke Trainer

**Affiliations:** 1 Department of Anesthesiology, Virginia Commonwealth University School of Medicine, Richmond, USA; 2 Department of Anesthesiology, Richmond Virginia Veterans Affairs Medical Center, Richmond, USA; 3 Department of Physical Medicine and Rehabilitation, Virginia Commonwealth University School of Medicine, Richmond, USA

**Keywords:** liposomal bupivacaine, adductor canal nerve block, postoperative pain, regional anesthesia, local anesthesia

## Abstract

Background

As total knee arthroplasty is one of the most common surgeries in the United States, it is important to identify regional anesthesia methods that optimize patient recovery. This study evaluates the effectiveness of adductor canal (AC) blocks with liposomal bupivacaine (LB) compared to other regional anesthesia techniques. We hypothesized that patients receiving single-shot (SS) AC blocks with LB would have lower postoperative opioid consumption compared to other groups.

Methods

A retrospective cohort analysis was conducted on patients from a single institution between January 2014 and December 2021. The primary outcome assessed was postoperative opioid use, with secondary outcomes including postoperative pain scores and hospital length of stay.

Results

The final analysis included 280 patients: 41 received an SS AC block with plain local anesthetic, 76 received a peripheral nerve catheter (PNC) with continuous ropivacaine, 79 received an SS AC block with LB, and 84 received no block. In fully adjusted models, postoperative opioid consumption on day one was significantly lower in the SS AC block with LB group compared to the no block group (b = 23.2, SE = 5.7, p < 0.0001), the PNC group (b = 15.5, SE = 5.7, p = 0.01), and the SS AC block with plain local anesthetic group (b = 18.9, SE = 6.9, p = 0.01). Additionally, hospital length of stay was significantly reduced in the LB group compared to the no block group (b = 1.5, SE = 0.3, p < 0.0001), the PNC group (b = 1.1, SE = 0.3, p < 0.0001), and the SS AC block with plain local anesthetic group (b = 1.5, SE = 0.3, p < 0.0001).

Conclusions

Patients who received an AC block with LB had higher pain scores on postoperative day 0 (POD0) but required less opioid medication on postoperative day 1 (POD1) and had a shorter hospital stay compared to patients who received other types of AC blocks or no block.

## Introduction

Total knee arthroplasty (TKA) is one of the most frequently performed surgical procedures in the United States, with a substantial increase in frequency expected in the coming decades. Nearly 500,000 TKAs were performed in 2019, and studies forecast a 139% increase in the number of TKAs performed by 2040 and a 469% increase by 2060 [1,2].

Anesthesiologists have explored various regional techniques to optimize postoperative analgesia while minimizing unwanted motor impairment [3-7]. Among these techniques, the adductor canal (AC) block has garnered attention for its efficacy in pain management post-TKA without the associated quadriceps weakness that can lead to increased falls and delayed postoperative ambulation, as seen with femoral nerve blocks [3,5-8].

Despite the consensus on the utility of AC blocks, debate persists regarding the preferred technique, particularly concerning continuous infusion via peripheral nerve catheter (PNC) versus a single-shot approach [9-12]. While continuous infusion of local anesthetic is often hypothesized to be superior, there is contradictory evidence regarding its impact on postoperative pain scores and opioid requirements [9-12].

An emerging adjunct to single-shot AC blocks is liposomal bupivacaine (LB), a long-acting formulation of bupivacaine that offers a prolonged duration of action compared to plain bupivacaine alone [13-17]. LB has recently been FDA-approved for AC blocks [18]. While existing literature suggests a reduction in postoperative opioid consumption with LB blocks, results regarding pain scores and hospital length of stay have varied [19-21]. Minimal studies have directly compared LB blocks to plain local anesthetic (PLA) blocks or to AC blocks with PNC.

Purpose

This study aims to compare SS AC blocks with LB to SS blocks with PLA, PLA with PNC infusion, and no nerve block in patients undergoing TKA. The primary outcome is postoperative opioid use, with secondary outcomes including hospital length of stay and postoperative pain scores.

## Materials and methods

A retrospective cohort study was conducted at the Central Virginia Veterans Affairs Health Care System after approval by the Institutional Review Board (study number 1572797-11) from January 2014 to December 2021. Data were collected through chart review, including patient demographics, medical history, type of regional anesthetic block received, postoperative opioid usage, median pain scores, use of non-opioid analgesics, and hospital length of stay. Patient clinical characteristics were used to compute a Charlson Comorbidity Index (CCI) (Table 1) [22].

**Table 1:** Patient Demographics and Outcomes Following Total Knee Arthroplasty *Significant overall differences using p < 0.05. ^†^Missing values. Numerical variables: Mean (SD). Categorical variables: N (%). N: number of patients, ASA: American Society of Anesthesiologists, CCI: Charlson Comorbidity Index, TIA/CVA: transient ischemic attack/cerebrovascular accident, OME: oral morphine equivalents, PLA: plain local anesthetic, SS: single shot, PNC: peripheral nerve catheter, LB: liposomal bupivacaine, LOS: length of stay, TKA: total knee arthroplasty, POD: postoperative day, PACU: post-anesthesia care unit, ERAS: Enhanced Recovery After Surgery.

	Overall	No Block	PNC	SS LB	SS PLA	P-value
	N = 280	N = 84 (30.0%)	N = 76 (27.14%)	N = 79 (28.21%)	N = 41 (14.64%)	
(i) Baseline patient characteristics						
Age	63.77 (8.69)	63.6 (9.2)	62.8 (8.7)	65.2 (8.4)	63.2 (8.2)	0.37
Gender						0.67
Male	255 (91.07%)	79 (94.05%)	69 (90.79%)	70 (88.61%)	37 (90.24%)	
Female	25 (8.93%)	5 (5.95%)	7 (9.21%)	9 (11.39%)	4 (9.76%)	
ASA						0.37
1	1 (0.36%)	0 (0%)	1 (1.32%)	0 (0%)	0 (0%)	
2	18 (6.43%)	4 (4.76%)	7 (9.21%)	6 (7.59%)	1 (2.44%)	
3	254 (90.7%)	79 (94.05%)	64 (84.21%)	71 (89.87%)	40 (97.56%)	
4	7 (2.5%)	1 (1.19%)	4 (5.26%)	2 (2.53%)	0 (0%)	
Charlson Comorbidity Index						0.32
Mild	130 (46.43%)	44 (52.38%)	38 (50%)	27 (34.18%)	21 (51.22%)	
Moderate	112 (40%)	30 (35.71%)	29 (38.16%)	39 (49.37%)	14 (34.15%)	
High	38 (13.57%)	10 (11.9%)	9 (11.84%)	13 (16.46%)	6 (14.63%)	
(ii) Comorbidities						
Coronary artery disease	58 (20.71%)	16 (19.05%)	17 (22.37%)	16 (20.25%)	9 (21.95%)	0.96
Hypertension	207 (73.93%)	64 (76.19%)	57 (75.0%)	58 (73.42%)	28 (68.29%)	0.81
Congestive heart failure	12 (4.29%)	3 (3.57%)	4 (5.26%)	4 (5.06%)	1 (2.44%)	0.89
Peripheral vascular disease	23 (8.21%)	9 (10.71%)	1 (1.32%)	11 (13.92%)	2 (4.88%)	0.02*
TIA/CVA	24 (8.57%)	7 (8.33%)	6 (7.89%)	8 (10.13%)	3 (7.32%)	0.95
Spinal cord injury	4 (1.43%)	0 (0.0%)	1 (1.32%)	1 (1.27%)	2 (4.88%)	0.12
Dementia	1 (0.36%)	0 (0.0%)	1 (1.32%)	0 (0.0%)	0 (0.0%)	0.42
Chronic kidney disease	39 (13.93%)	10 (11.90%)	16 (21.05%)	9 (11.39%)	4 (9.76%)	0.21
Diabetes mellitus	90 (32.14%)	23 (27.38%)	26 (34.21%)	27 (34.18%)	14 (34.15%)	0.74
(iii) Additional analgesics						
Acetaminophen	238 (85%)	68 (80.95%)	64 (84.21%)	78 (98.73%)	28 (68.29%)	<0.0001*
Patient Controlled Analgesia	47 (16.79%)	4 (4.76%)	34 (44.74%)	1 (1.27%)	8 (19.51%)	<0.0001*
Gabapentin/Pregabalin	143 (51.07%)	25 (29.76%)	47 (61.84%)	53 (67.09%)	18 (43.90%)	<0.0001*
(iv) Outcomes following TKA						
OME 0	20.92 (21.67)	25.79 (22.95)	15.8 (19.39)	24.06 (18.73)	14.39 (25.08)	0.0028*
OME 1	54.13 (39.71)	66.82 (39.48)	53.93 (35.13)	36.91 (28.52)	61.67 (53.66)	<0.0001*
Median Pain Score 0^†^	4.07 (2.59)	4.82 (2.76)	3.25 (2.41)	4.30 (2.23)	3.59 (2.79)	0.0009*
Median Pain Score 1^†^	4.67 (2.13)	4.95 (2.07)	4.26 (1.91)	4.69 (2.06)	4.85 (2.66)	0.21
LOS	3.24 (1.84)	3.7 (1.7)	3.2 (1.3)	2.3 (2.0)	3.9 (1.8)	<0.0001*

All patients were veterans. Inclusion criteria consisted of age greater than 18 years at the time of total knee arthroplasty and classification as I-IV via the American Society of Anesthesiologists’ (ASA) physical classification scale. Patients must have also received either no block, an SS AC block with PLA, an SS AC block with LB, or an AC block with a PNC. Patients with a history of opioid use disorder, opioid dependence, or those who received repeat peripheral nerve blocks, failed initial blocks, or were classified as ASA class V-VI were excluded from the study. All narcotic medications were converted and recorded as oral morphine equivalents (OMEs). Pain scores were obtained by nursing staff, having patients rate their pain on a visual analog scale (VAS) [23]. OMEs and pain scores were collected from the immediate postoperative period through discharge. Postoperative day (POD) zero began upon discharge from the PACU to the surgical floor, and each POD ended at midnight. Daily opioid use and pain scores were recorded through postoperative day three, although some patients required shorter or longer hospital stays. The primary outcome of this study was postoperative OME requirements. Secondary outcomes included hospital length of stay and postoperative pain scores.

The treatment groups were divided into four categories: no block, AC block with PNC, SS AC block with LB, and SS AC block with PLA. We compared patient sociodemographic factors (age, gender), clinical characteristics (ASA, CCI), use of non-opioid analgesics (acetaminophen, gabapentinoids), and outcomes (amount of postoperative opioid use assessed via OMEs, hospital length of stay, median pain scores) across the four patient groups. Chi-square tests were used for categorical variables and ANOVA for continuous variables to examine differences between the groups. Five linear regression models were constructed for outcomes, adjusting for the covariates and respective median pain scores, with the SS LB block group as the reference for comparisons. All statistical analyses were performed using Python and SAS 9.4 (SAS Institute Inc., Cary, North Carolina) analytical programs. A significance value of 0.05 was used for all statistical analyses.

## Results

The final analysis included 272 patients after adjusting for missing data on covariates. There was no difference in the sociodemographic factors, including age, gender, ASA classification, and CCI (Table 1).

**Figure 1:**
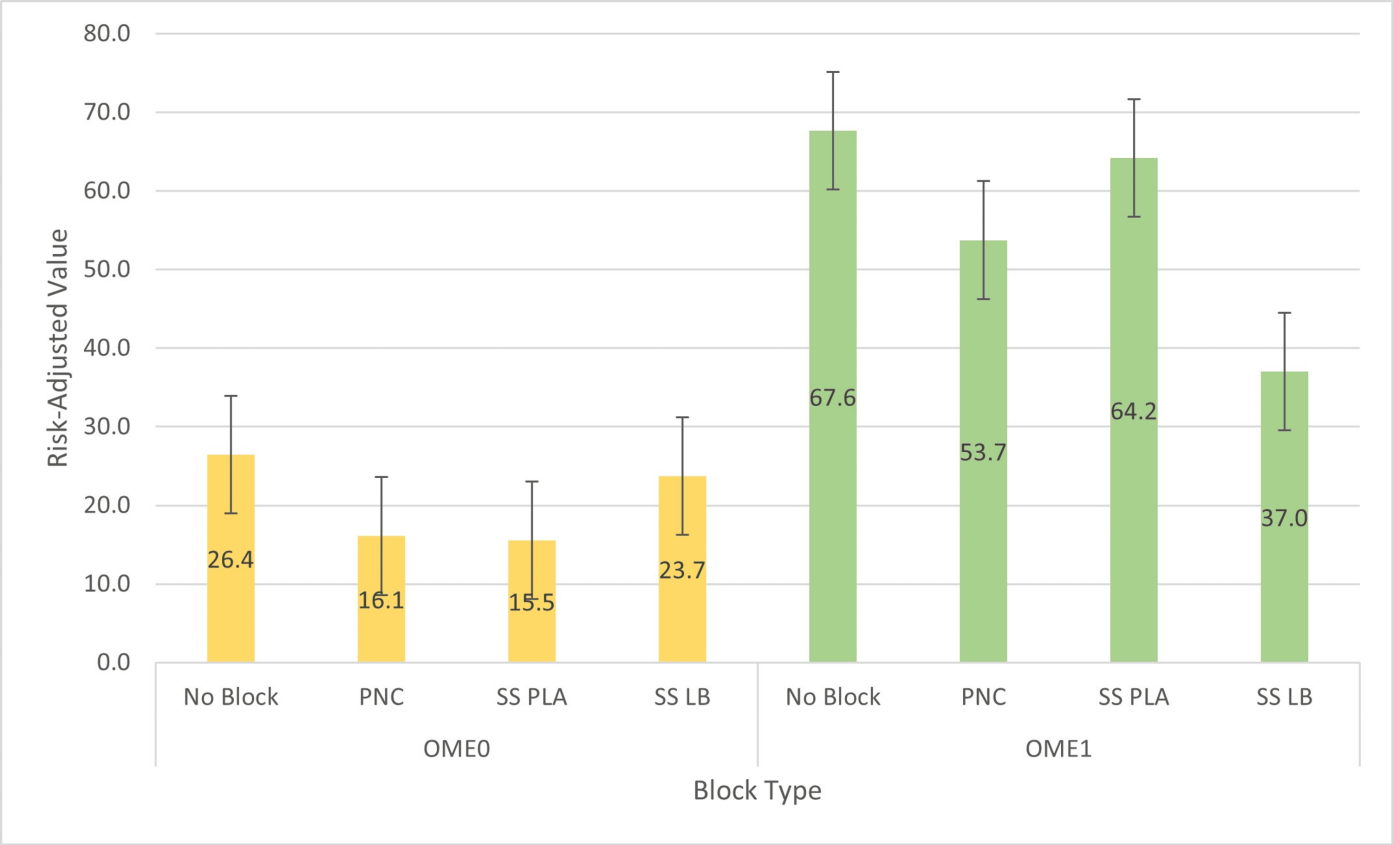
Average risk-adjusted value of OME0 and OME1 by treatment groups OME0: oral morphine equivalents on postoperative day zero, OME1: oral morphine equivalents on postoperative day one.

All the adjusted models were controlled for age, gender, ASA, CCI, and LOS. Of these patients, 79 received an SS AC block with LB, 41 received an SS AC block with PLA, 76 received an AC block with PNC, and 84 received no peripheral nerve block. For postoperative day one opioid use, relative to the SS LB group, we found significantly higher OME for no block (b = 23.2, SE = 5.7, p < 0.0001), for PNC (b = 15.5, SE = 5.7, p = 0.01), and for SS PLA (b = 18.9, SE = 6.9, p = 0.01) (Table 2, Figure 1).

**Table 2:** Linear Regression Models for Primary and Secondary Outcomes Following Total Knee Arthroplasty *Significant differences using p < 0.05. Block SS liposomal bupivacaine, CCI mild, and gender male were used as reference categories. OME0: oral morphine equivalents on postoperative day zero, OME1: oral morphine equivalents on postoperative day one, LOS: length of stay, PNC: peripheral nerve catheter, SS PLA: single shot with plain local anesthetic, SE: standard error, ASA: American Society of Anesthesiologists, CCI: Charlson Comorbidity Index, NA: not applicable.

	OME0	OME1	LOS	Median Pain Score POD0	Median Pain Score POD1
Unconditional models					
No block	2.7 (SE = 3.4, p = 0.43)	30.6 (SE = 6.0, p < 0.0001*)	1.4 (SE = 0.3, p < 0.0001*)	0.5 (SE = 0.4, p = 0.18)	0.3 (SE = 0.3, p = 0.38)
Block PNC	-7.6 (SE = 3.4, p = 0.03*)	16.7 (SE = 6.2, p = 0.01*)	0.9 (SE = 0.3, p = 0.0011*)	-1.0 (SE = 0.4, p = 0.01*)	-0.4 (SE = 0.3, p = 0.19)
Block SS PLA	-8.2 (SE = 4.2, p = 0.05)	27.2 (SE = 7.6, p = 0.0004*)	1.5 (SE = 0.3, p < 0.0001*)	-0.7 (SE = 0.5, p = 0.17)	0.2 (SE = 0.4, p = 0.63)
Adjusted models					
No block	0.8 (SE = 3.2, p = 0.82)	23.2 (SE = 5.7, p < 0.0001*)	1.5 (SE = 0.3, p < 0.0001*)	0.5 (SE = 0.4, p = 0.21)	0.2 (SE = 0.3, p = 0.53)
Block PNC	-5.3 (SE = 3.2, p = 0.11)	15.5 (SE = 5.7, p = 0.01*)	1.1 (SE = 0.3, p < 0.0001*)	-1.1 (SE = 0.4, p = 0.006*)	-0.6 (SE = 0.3, p = 0.10)
Block SS PLA	-7.2 (SE = 3.9, p = 0.07)	18.9 (SE = 6.9, p = 0.01*)	1.5 (SE = 0.3, p < 0.0001*)	-0.8 (SE = 0.5, p = 0.09)	0.1 (SE = 0.4, p = 0.80)
Median Pain Score	2.9 (SE = 0.5, p < 0.0001*)	7.3 (SE = 1.0, p < 0.0001*)	0.1 (SE = 0.0, p = 0.01*)	NA	NA
Age	-0.8 (SE = 0.2, p < 0.0001*)	-1.1 (SE = 0.3, p = 0.001*)	0.1 (SE = 0.0, p = 0.0004*)	-0.1 (SE = 0.0, p = 0.004*)	-0.1 (SE = 0.0, p = 0.003*)
Gender	-6.8 (SE = 4.2, p = 0.10)	-6.9 (SE = 7.3, p = 0.34)	0.9 (SE = 0.4, p = 0.01*)	0.8 (SE = 0.5, p = 0.16)	-0.4 (SE = 0.4, p = 0.38)
ASA	10.6 (SE = 3.8, p = 0.01*)	6.5 (SE = 6.7, p = 0.33)	0.5 (SE = 0.3, p = 0.15)	0.2 (SE = 0.5, p = 0.66)	0.1 (SE = 0.4, p = 0.82)
CCI high	6.9 (SE = 4.4, p = 0.12)	6.3 (SE = 7.7, p = 0.41)	0.1 (SE = 0.4, p = 0.82)	0.5 (SE = 0.6, p = 0.40)	-0.2 (SE = 0.5, p = 0.75)
CCI moderate	0.8 (SE = 2.9, p = 0.79)	-5.2 (SE = 5.1, p = 0.31)	-0.3 (SE = 0.3, p = 0.20)	0.1 (SE = 0.4, p = 0.82)	0.3 (SE = 0.3, p = 0.33)
LOS	-0.7 (SE = 0.7, p = 0.31)	1.8 (SE = 1.2, p = 0.15)	NA	(-)	(-)

**Figure 2:**
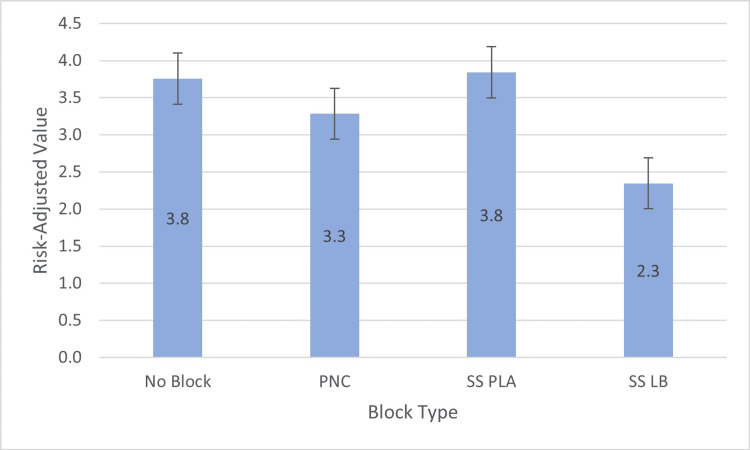
Average risk-adjusted value of hospital length of stay (LOS) by treatment groups PNC: peripheral nerve catheter, SS PLA: single shot with plain local anesthetic, SS LB: single shot with liposomal bupivacaine.

**Figure 3:**
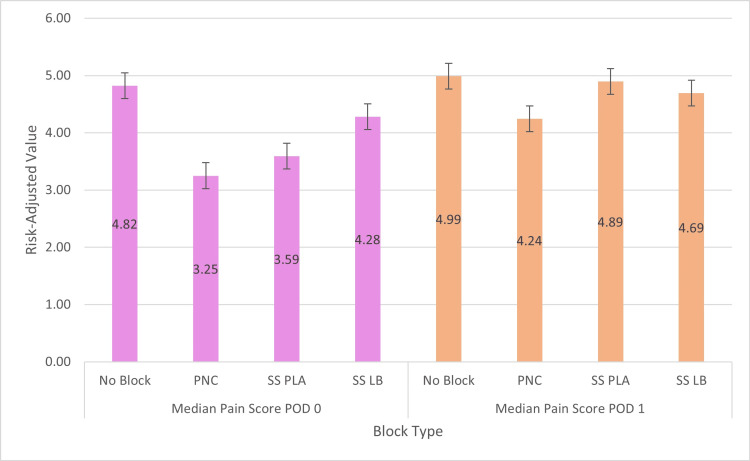
Average risk-adjusted value of median pain score on postoperative day (POD) 0 and POD 1 by treatment groups PNC: peripheral nerve catheter, SS PLA: single shot with plain local anesthetic, SS LB: single shot with liposomal bupivacaine.

For LOS, relative to the SS LB group, we found significantly higher LOS for no block (b = 1.5, SE = 0.3, p < 0.0001), for PNC (b = 1.1, SE = 0.3, p < 0.0001), and for SS PLA (b = 1.5, SE = 0.3, p < 0.0001) (Table 2, Figure 2). For median pain scores on postoperative day zero, relative to the SS LB group, we found a significant reduction in pain scores only for PNC (b = -1.1, SE = 0.4, p = 0.01) (Table 2, Figure 3). No differences were found in opioid requirements on postoperative day zero and median pain scores on postoperative day one (Tables 1, 2).

We have intentionally excluded LOS as a covariate in the pain score models to prevent the misinterpretation of reverse causality, based on the understanding that while pain scores may influence LOS, the reverse is less likely. Including LOS could lead to biased estimates.

## Discussion

This retrospective study showed that the use of LB in AC blocks for patients undergoing TKA resulted in significantly lower inpatient opioid requirements on POD1 compared to patients receiving no block, PNC, or SS PLA. No difference existed between the groups on POD0.

For the secondary outcomes, the LB group had significantly higher pain scores on POD0 compared to the other groups. No difference existed between the groups on POD1. The LB group also had a significantly lower LOS compared to the other groups.

This study adds to the growing body of evidence investigating the optimal regional technique for patients undergoing TKA. Our results surrounding the use of LB in AC blocks mirror the heterogeneity found throughout this body of work. The lack of difference in opioid use and increased pain scores on POD0 for patients receiving LB is expected given the decreased amount of plain local anesthetic administered with the primary block. The patients in the PNC and SS PLA groups received only plain local anesthetic in their AC block, resulting in a denser block with more immediate analgesia. When using LB, the operator must mix the LB with plain bupivacaine, with a maximum of a 1:2 ratio [24]. Therefore, while LB provides prolonged analgesia, it comes at the expense of producing a less dense block and decreased upfront analgesia. This finding aligns with patient experiences observed by members of the regional anesthesia team at our institution.

Despite the increased pain on POD0, the patients receiving LB appeared to benefit from its extended analgesia, as evidenced by decreased opioid requirements on POD1. While the LB continued to provide analgesia on POD1, the block in patients in the SS PLA group had worn off. The differences observed in the PNC group may be explained by incorrectly placed catheters or catheters that became dislodged during the inpatient stay. Not only is LB easier and faster to administer than a PNC, but the lower opioid requirements on POD1 suggest that LB may be the superior option for prolonged analgesia.

LOS was the final secondary outcome analyzed, showing that patients receiving LB had a significantly shorter stay than those in the other groups. Improved pain control with regional anesthesia can affect discharge readiness by facilitating physical therapy, decreasing reliance on intravenous pain medications, and more. However, interpreting this result is challenging due to the many confounding factors that influence patient discharge. This retrospective study spanned an extended period during which the regional team initially performed more AC blocks with PLA and PNC but gradually transitioned to more SS blocks with LB. Simultaneously, the hospital placed a greater emphasis on early discharge and ERAS protocols. The change in patient care and the shift towards different discharge goals likely explain this result more than any particular regional technique.

Despite these positive outcomes observed with AC blocks with LB, it is crucial to interpret these findings within the context of the study's limitations. The primary limitation stems from the retrospective nature of the study over a seven-year period, which makes it challenging to account for confounding factors related to changes in clinical practice over time. Particularly notable are the incorporation of ERAS pathways and shifts in narcotic prescription patterns, both of which likely influenced outcomes. Additionally, the study's exclusive focus on veterans from a large VA hospital introduces limitations in terms of generalizability to other patient populations and healthcare settings. While the unique patient demographics at the VA hospital provide valuable insights, the results may not be applicable to patients undergoing TKA in outpatient private orthopedic practices due to potential differences in care delivery and patient characteristics.

Future directions include larger randomized controlled studies comparing these groups, not only for acute outcomes but also to explore long-term impacts such as chronic pain and opioid addiction. Additionally, collecting data on early mobilization and physical therapy progress may provide further insights into the factors influencing LOS between groups.

## Conclusions

In this single-center, retrospective review, we compared AC blocks with liposomal bupivacaine, plain local anesthetic, continuous peripheral nerve catheter, and no nerve block in 272 patients undergoing TKA. We found that patients undergoing TKA who received AC blocks with liposomal bupivacaine had significantly lower inpatient opioid requirements on postoperative day one compared to those who received plain local anesthetic, continuous peripheral nerve catheter, or no nerve block. No difference in opioid requirements existed between the groups on postoperative day zero. For our secondary outcomes, patients who received AC blocks with liposomal bupivacaine had significantly higher pain scores on postoperative day zero compared to the other groups. However, no difference in pain scores existed between the groups on postoperative day one. It is crucial to interpret these findings within the context of the study's limitations, and further investigative research is required.
